# Stanniocalcin-1 Protects a Mouse Model from Renal Ischemia-Reperfusion Injury by Affecting ROS-Mediated Multiple Signaling Pathways

**DOI:** 10.3390/ijms17071051

**Published:** 2016-07-12

**Authors:** Dajun Liu, Huiping Shang, Ying Liu

**Affiliations:** Department of Nephrology, Shengjing Affiliated Hospital of China Medical University, Shenyang 110036, China; songxs1981@sina.com (H.S.); yingliu1@163.com (Y.L.)

**Keywords:** stanniocalcin-1, renal ischemia-reperfusion injury, apoptosis signal-regulating kinase 1, p-IkB kinase, extracellular signal-regulated kinase, protein kinase C, p-NF-κB, caspase-3, reactive oxygen species-mediated pathways, phosphorylated mitogen-activated protein kinase kinase

## Abstract

Stanniocalcin-1 (STC-1) protects against renal ischemia-reperfusion injury (RIRI). However, the molecular mechanisms remain widely unknown. STC-1 inhibits reactive oxygen species (ROS), whereas most ROS-mediated pathways are associated with ischemic injury. Therefore, to explore the mechanism, the effects of STC-1 on ROS-medicated pathways were studied. Non-traumatic vascular clamps were used to establish RIRI mouse models. The serum levels of STC-1, interleukin-6 (IL-6), interferon (IFN) γ, P53, and capase-3 were measured by ELISA kits. Superoxide dismutase (SOD) and malondialdehyde (MDA) were measured by fluorescence spectrofluorometer. All these molecules changed significantly in a RIRI model mouse when compared with those in a sham control. Kidney cells were isolated from sham and model mice. STC-1 was overexpressed or knockout in these kidney cells. The molecules in ROS-medicated pathways were measured by real-time quantitative PCR and Western blot. The results showed that STC-1 is an effective ROS scavenger. The serum levels of STC-1, MDA and SOD activity were increased while the serum levels of IL-6, iIFN-γ, P53, and capase-3 were decreased in a model group when compared with a sham control (*p* < 0.05). Furthermore, the levels of STC-1,p53, phosphorylated mitogen-activated protein kinase kinase (p-MEKK-1), c-Jun N-terminal kinase (p-JNK), extracellular signal-regulated kinase (p-ERK), IkB kinase (p-IKK), nuclear factor (NF) κB, apoptosis signal-regulating kinase 1 (ASK-1) and caspase-3 changed significantly in kidney cells isolated from a RIRI model when compared to those isolated from a sham control (*p* < 0.05). Meanwhile, STC-1 overexpression or silence caused significant changes of the levels of these ROS-mediated molecules. Therefore, STC-1 maybe improve anti-inflammation, anti-oxidant and anti-apoptosis activities by affecting ROS-mediated pathways, especially the phospho-modifications of the respective proteins, resulting in the increase of SOD and reduce of capase-3, p53, IL-6 and IFN-γ.

## 1. Introduction

Ischemia/reperfusion renal injury (RIRI) has been characterized with restricted blood flow to kidney and then restores the normal blood flow and oxygen supply. Renal injury often occurs after kidney infarction [1], kidney sepsis [2] and kidney transplantation [3]. The situation will exacerbate renal damage by affecting inflammatory activities of various cytokines and the production of reactive oxygen species (ROS) [4], cellular function [5], inflammatory responses [6], and cellular apoptosis [7], which are the main factors for the pathogenesis of RIRI. RIRI is a clinical symptom with renal dysfunction and high mortality rates [8]. The pathogenesis of RIRI is complicated and hard to be treated. It is necessary to understand the more molecular mechanisms for causing the RIRI or preventing the damage caused by RIRI.

Stanniocalcin-1 (STC-1) exists in various tissues and has been regarded as autocrine or paracrine factor [9]. STC-1 is an important regulator for kidney and intestinal calcium transport, which can affect cellular metabolism and calcium homeostasis [10]. STC-1 is expressed in many cells and can be released into extracellular milieu [11], and binds the proteins from cell surface, and also interacts mitochondrial membrane [12,13].

STC-1 can suppress superoxide generation [14], inflammation and apoptosis [15] in mammalians. Much evidence suggests that STC-1 has potent anti-inflammatory and protective functions for RIRI [16,17], especially for ischemic tolerance [18]. According to a previous report, STC-1 is critical elements for activation of AMP-activated protein kinase (AMPK) activity in kidney. AMPK regulates STC-1-induced expression of UCP2 and sirtuin 3, and shows protecting functions for RIRI [16]. Another report showed that STC-1 reduced ROS production and caused higher ratio of activated extracellular regulated kinase (ERK)/activated Jun-N-terminal kinase (JNK) [17]. ROS can be associated with many different pathways, suggesting that there are other molecular mechanisms that need to be explored. In response to ischemic condition caused by RIRI, kidney shows protective functions for subsequent ischemic stresses. Apoptosis signaling kinase-1 [19], protein kinase C (PKC) [20], ERK [21], p53 [22], IkB kinase (IKK) [23], mitogen-activated protein kinase kinase (MEKK-1) [24], JNK [25], caspase-3 [26] and nuclear factor NF-κB [27] are associated with oxygen homeostasis and ischemic injury. All of them are associated with ROS-mediated pathways [28,29,30,31], whereas the overexpression of STC-1 has been reported to inhibit ROS production [32]. Furthermore, phosphate-modification plays an important role in the most molecules. For example, the inhibition of phosphate-modification of JNK (p-JNK) ameliorated myocardial I/R injury [33]. Another example, cardioprotective effects were associated with the levels of p-ERK [34]. The role phosphate modification of these important proteins was seldom reported in RIRI. Therefore, we investigated the effects of STC-1 on the main molecules ASK-1, p-ERK, ERK, PKC, p53, p-IKK, IKK, p-MEKK-1, MEKK, p-JNK, JNK, caspase-3 and nuclear factor NF-κB. Meanwhile, anti-oxidant, anti-inflammation and anti-apoptosis activities were also investigated by measuring the levels of superoxide dismutase (SOD), malondialdehyde (MDA), interleukin-6 (IL), interferon-γ (IFN-γ) and p53.

## 2. Results

### 2.1. Evaluation for Renal Dysfunction of RIRI Model

**Figure 1:**
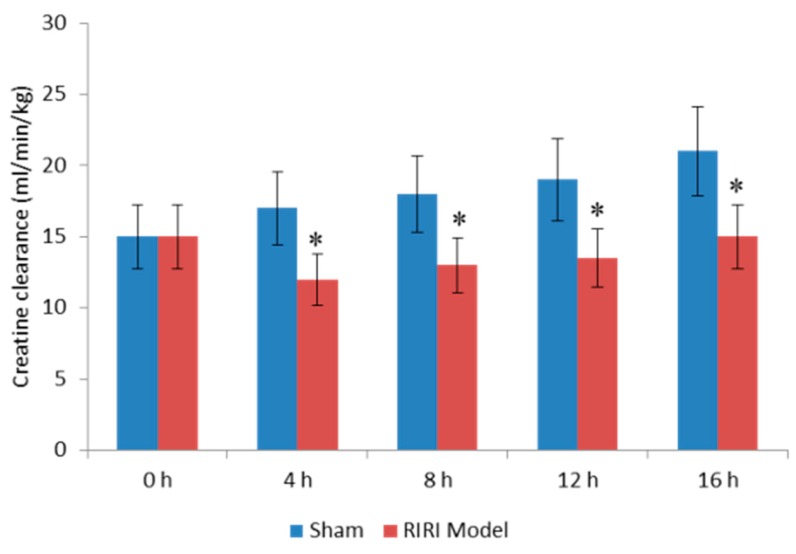
Creatinine clearance rate in mice. RIRI, Renal ischemia-reperfusion injury. All data were presented as mean values ± S.D. *n* = eight in each group. * *p* < 0.05 compared to the sham group.

Renal function was evaluated by measuring the changes of serum creatinine after establishment of RIRI model. The results showed that serum creatinine clearance was higher in subsequent period than before surgery. However, there was no significantly statistic difference for the changes in the same group. Comparatively, as seen in Figure 1, the creatinine clearance was significantly reduced in RIRI mice models when compared with the sham controls after four-hour establishment of RIRI model (*p* < 0.05) (Figure 1).

### 2.2. Increased Expression of STC-1 in the Kidney of Mouse Model

ELISA analysis showed that the average serum levels of STC-1 were 6.88 ± 1.56 ng/mL in a MSG group and 14.96 ± 3.24 ng/mL in a MG group (*p* = 0.002). Serum levels of STC-1 were increased in RIRI models when compared with sham controls. The result suggested that STC-1 functionally participated in physiological activity after the establishment of RIRI model.

Comparatively, the average values of serum STC-1 were 9.34 ± 2.18 ng/mL and 19.22 ± 4.58 ng/mL in SSG and MSG groups, which were significantly higher than the groups without STC-1 transfection (*p* = 0.001). The average values of serum STC-1 were 4.76 ± 1.09 ng/mL and 5.41 ± 1.12 ng/mL in SSShG and MSSG groups, respectively, which were significantly lower than the groups without STC-1 transfection (*p* = 0.001). All of these results suggested that the mice were successfully transfected with the vector with STC-1 gene or STC-1 shRNA.

### 2.3. The Effects of STC-1 on Immunological and Biochemical Parameters

**Figure 2:**
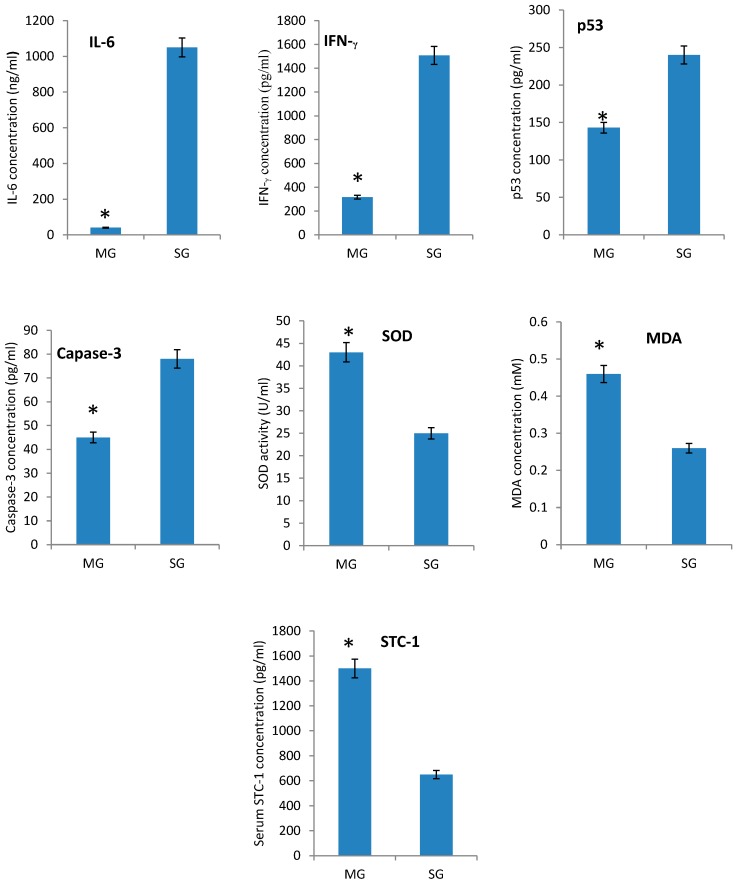
The serum biochemical and immunological parameters in different treated mice: (**A**) the serum protein levels of IL-6; (**B**) the serum protein levels of IFN-γ; (**C**) the serum protein levels of p53; (**D**) the serum protein levels of caspase-3; (**E**) the serum activity of SOD; (**F**) the serum protein levels of MDA; and (**G**) the serum protein levels of STC-1. Among 16 mice, eight mice were used to create RIRI models and another eight mice were used as a sham group. All data were presented as mean values ± S.D. * *p* < 0.05 compared to the model group.

Meanwhile, the serum levels of inflammatory cytokines IL-6 and IFN-γ also decreased in a model group when compared with a sham group (Figure 2A,B, *p* < 0.05) while more inflammatory cytokines IL-6 and IFN-γ will make renal injury worse [23,35]. In contrast, the apoptotic factors, the serum levels of p53 and capase-3 decreased in a model group when compared with a sham group (Figure 2C,D, *p* < 0.05). ROS plays an important role in the pathogenesis of RIRI [36]. To protect against RIRI, SOD activity was improved, and serum levels of MDA increased in RIRI models when compared with Sham controls (Figure 2E,F, *p* < 0.05).

Comparatively, the serum level of STC-1 was higher in a model group than in a sham group (Figure 2G, *p* < 0.05). The serum levels of SOD and MDA increased (Figure 2E,F, *p* < 0.05). In contrast, the levels of IL-6, IFN-γ, p53 and caspase-3 were decreased in a model group (Figure 2A–D, *p* < 0.05). All of these findings might suggest that STC-1 plays an important role for protecting mice from RIRI by controlling the oxidant apoptosis and inflammatory responses by affecting the activities of SOD, MDA, p53, caspase-3, IL-6 and IFN-γ.

### 2.4. Intercellular ROS Concentration

**Figure 3:**
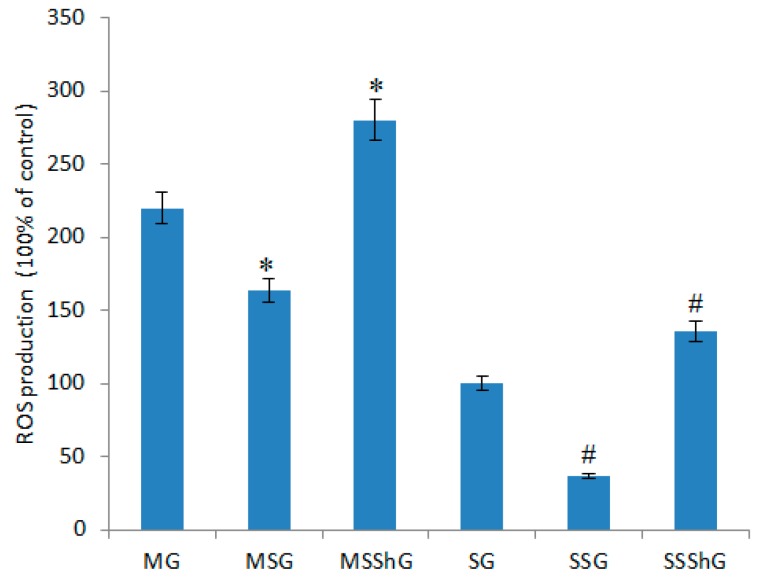
ROS production in different cells. Among 16 mice, eight mice were used to create RIRI models as a model group and eight mice were used as a sham group. Renal progenitor cells were isolated from the kidney cortex of RIRI model and sham mice after 16-h surgery. For the kidney cells isolated from eight model mice, they were further subdivided into three groups based on different treatment options (model group (MG); STC-1 expression group (MSG), the mice were transfected with *STC-1* gene to overexpress STC-1; and STC-1 shRNA group (MSShG), the mice were transfected with STC-1 shRNA to knockdown STC-1). In the same way, the kidney cells from eight sham mice were subdivided into another three groups based on different treatment options sham groups (sham group (SG); STC-1 expression group (SSG), the mice were transfected with *STC-1* gene to overexpress STC-1; and STC-1 shRNA group (SSShG), the mice were transfected with STC-1 shRNA to knockdown STC-1). The ROS levels were measured from all the cells cultured for 24 h. All data were presented as the mean values ± S.D. * *p* < 0.05 via a model group and ^#^ *p* < 0.05 via a sham group.

ROS levels were significantly increased (to 120%) when compared to the concentrations in the cells isolated from sham mice (*p* < 0.05, Figure 3). The overexpression of STC-1 can significantly reduce ROS production in the cells isolated from model and sham mice (*p* < 0.05, Figure 3). In contrast, the STC-1 silence increased ROS levels (*p* < 0.05, Figure 3). Taken together, these results suggest that STC-1 is an effective ROS scavenger.

### 2.5. STC-1 Affects the mRNA Levels of ROS-Mediated Molecules

**Figure 4:**
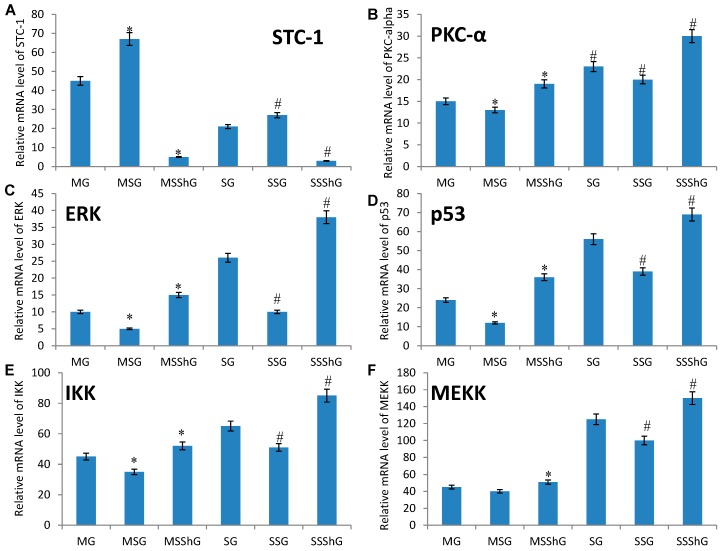
The effects of STC-1 on the mRNA levels of ROS-mediated molecules. Among 16 mice, eight mice were used to create RIRI models as a model group and eight mice were used as a sham group. Renal progenitor cells were isolated from the kidney cortex of RIRI model and sham mice after 16-h surgery. For the kidney cells isolated from eight model mice, they were further subdivided into three groups based on different treatment options (model group (MG); STC-1 expression group (MSG), the mice were transfected with *STC-1* gene to overexpress STC-1; and STC-1 shRNA group (MSShG), the mice were transfected with STC-1 shRNA to knockdown STC-1). In the same way, the kidney cells from 8 sham mice were subdivided into another three groups based on different treatment options (sham group (SG); STC-1 expression group (SSG), the mice were transfected with STC-1 gene to overexpress STC-1; and STC-1 shRNA group (SSShG), the mice were transfected with STC-1 shRNA to knockdown STC-1). The mRNA levels were measured from the same part of renal tissues obtained after 4-h surgery: (**A**) the changes of mRNA levels of STC-1 in different groups; (**B**) the changes of mRNA levels of PKC-α in different groups; (**C**) the changes of mRNA levels of ERK in different groups; (**D**) the changes of mRNA levels p53 in different groups; (**E**) the changes of mRNA levels of IKK in different groups; (**F**) the changes of mRNA levels of MEKK-1 in different groups; (**G**) the changes of mRNA levels of JNK in different groups; (**H**) the changes of mRNA levels of ASK-1 in different groups; (**I**) the changes of mRNA levels of caspase-3 in different groups; and (**J**) the changes of mRNA levels of NF-κB in different groups. All data were normalized to actin, and presented as the mean values ± S.D. * *p* < 0.05 compared to the model group and ^#^ *p* < 0.05 compared to the sham group.

Among 16 mice, eight mice were used to create RIRI models as a model group and another eight mice were used as a sham group. Renal progenitor cells were isolated from the kidney cortex of RIRI model and sham mice after 16-h surgery. For the kidney cells isolated from eight model mice, they were further subdivided into three groups based on different treatment options (model group (MG); STC-1 expression group (MSG), the mice were transfected with STC-1 gene to overexpress STC-1; and STC-1 shRNA group (MSShG), the mice were transfected with STC-1 shRNA to knockdown STC-1). In the same way, the kidney cells from eight sham mice were subdivided into another three groups based on different treatment options (sham group (SG); STC-1 expression group (SSG), the mice were transfected with STC-1 gene to overexpress STC-1; and STC-1 shRNA group (SSShG), the mice were transfected with STC-1 shRNA to knockdown STC-1). Present results showed that mRNA level of STC-1 increased when compared with a sham group (Figure 4A, *p* < 0.05). The mRNA level of STC-1 increased in MSG and MSSG groups and decreased in SSG and SSShG groups (Figure 4A, *p* < 0.05), suggested that the cells were successfully transfected with the constructed vectors, STC-1 and or STC-1 shRNA.

In contrast, the mRNA level of PKC-α (Figure 4B), ERK (Figure 4C), p53 (Figure 4D), IKK (Figure 4E), MEKK (Figure 4F), JNK (Figure 4G), ASK-1 (Figure 4H), caspase-3 (Figure 4I), and NF-κB (Figure 4J) decreased when compared with a sham group (*p* < 0.05). The overexpression of STC-1 decreased the mRNA level of PKC-α (Figure 4B), ERK (Figure 4C), p53 (Figure 4D), IKK (Figure 4E), MEKK (Figure 4F), JNK (Figure 4G), ASK-1 (Figure 4H), caspase-3 (Figure 4I), and NF-κB (Figure 4J) in MSG and SSG groups. On the other hand, STC-1 silence increased mRNA levels of PKC-α (Figure 4B), ERK (Figure 4C), p53 (Figure 4D), IKK (Figure 4E), MEKK (Figure 4F), JNK (Figure 4G), ASK-1 (Figure 4H), caspase-3 (Figure 4I), and NF-κB (Figure 4J) in MSShG and SSShG groups (*p* < 0.05), suggested STC-1 reduces the mRNA level of PKC-α (Figure 4B), ERK (Figure 4C), p53 (Figure 4D), IKK (Figure 4E), MEKK (Figure 4F), JNK (Figure 4G), ASK-1 (Figure 4H), caspase-3 (Figure 4I), and NF-κB (Figure 4J).

### 2.6. STC-1 Decreased the Protein Levels of ROS-Mediated Molecules

**Figure 5:**
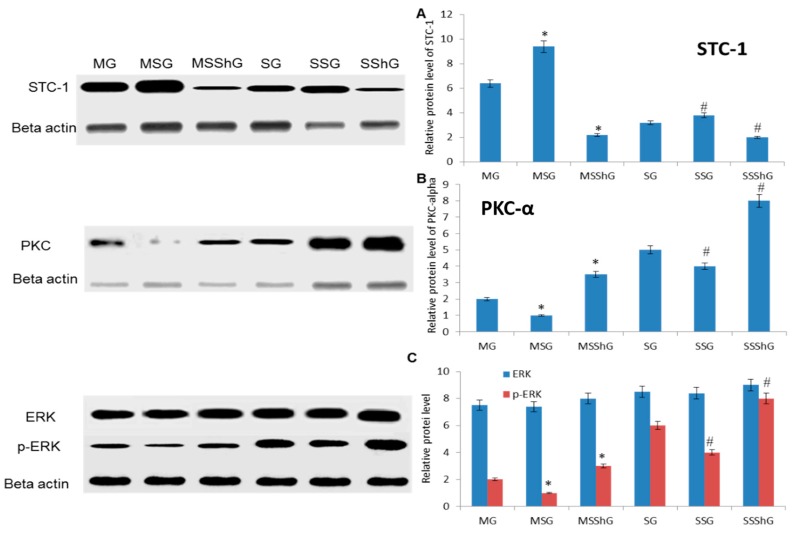

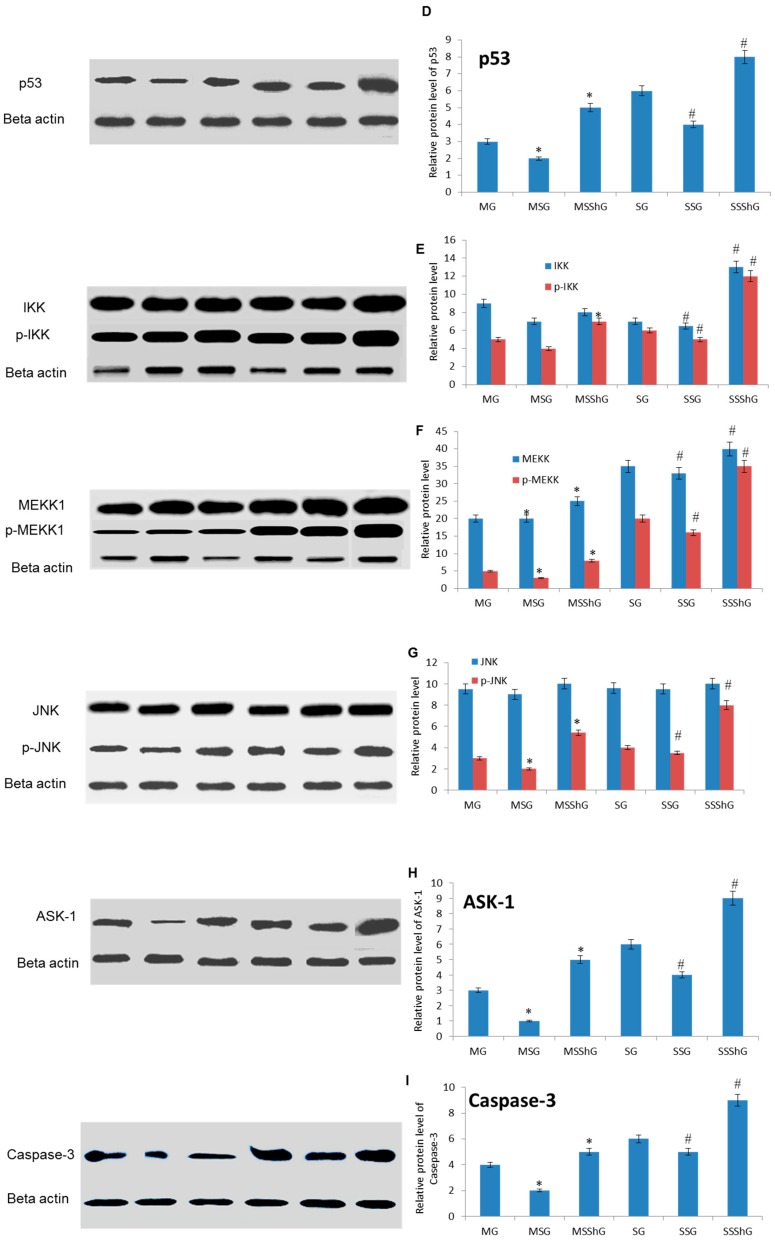

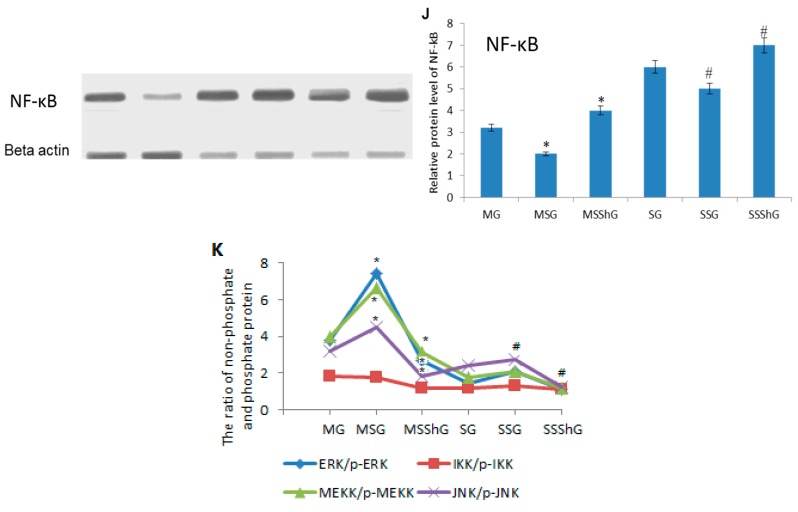
The effects of STC-1 on the protein levels of PKC-α, p-IKK, p-MEKK-1, p-NF-κB and caspase-3. Among 16 mice, eight mice were used to create RIRI models as a model group and eight mice were used as a sham group. Renal progenitor cells were isolated from the kidney cortex of RIRI model and sham mice after 16-h surgery. For the kidney cells isolated from eight model mice, they were further subdivided into three groups based on different treatment options (model group (MG); STC-1 expression group (MSG), the mice were transfected with *STC-1* gene to overexpress STC-1; and STC-1 shRNA group (MSShG), the mice were transfected with STC-1 shRNA to knockdown STC-1). In the same way, the kidney cells from 32 sham mice were subdivided into another three groups based on different treatment options (sham group (SG); STC-1 expression group (SSG), the mice were transfected with *STC-1* gene to overexpress STC-1; and STC-1 shRNA group (SSShG), the mice were transfected with STC-1 shRNA to knockdown STC-1). The mRNA levels were measured from the same part of renal tissues obtained after 4-h surgery: (**A**) the changes of protein levels of STC-1 in different groups; (**B**) the changes of protein levels of PKC-α in different groups; (**C**) the changes of protein levels of p-ERK in different groups; (**D**) the changes of protein levels p53 in different groups; (**E**) the changes of protein levels of p-IKK in different groups; (**F**) the changes of protein levels of p-MEKK-1 in different groups; (**G**) the changes of protein levels of p-JNK in different groups; (**H**) the changes of protein levels of ASK-1 in different groups; (**I**) the changes of protein levels of caspase-3 in different groups; (**J**) the changes of protein levels of NF-κB in different groups; and (**K**) the ratio of non-phosphate proteins and phosphate proteins. All data were normalized to actin, and presented as the mean values ± S.D. * *p* < 0.05 via model group and # *p* < 0.05 via sham group.

Just like the results from qRT-PCR, our results also showed that protein level of STC-1 increased after 4-h RIRI surgery when compared with that from a sham group (Figure 5A, *p* < 0.05). The protein level of STC-1 increased in MSG and SSG groups and decreased in SSG and SSShG groups (Figure 5A, *p* < 0.05), suggested the changes of mRNA levels results in the corresponding changes of protein levels of STC-1 in the mice.

In contrast, protein levels of PKC-α (Figure 5B), p-ERK, ERK (Figure 5C), p53 (Figure 5D), IKK (Figure 5E), p-MEKK, MEKK (Figure 5F), p-JNK (Figure 5G), ASK-1 (Figure 5H), caspase-3 (Figure 5I), and NF-κB (Figure 5J) decreased in the cells isolated from RIRI model when compared with the cells isolated from a sham group (*p* < 0.05). The overexpression of STC-1 decreased the protein level of PKC-α (Figure 5B), p-ERK, ERK (Figure 5C), p53 (Figure 5D), p-IKK, IKK (Figure 5E), p-MEKK, MEKK (Figure 5F), p-JNK, JNK (Figure 5G), ASK-1 (Figure 5H), caspase-3 (Figure 5I), and NF-κB (Figure 5J) in MSG and MSSG groups. On the other hand, STC-1 silence increased the protein levels of PKC-α (Figure 5B), p-ERK (Figure 5C), p53 (Figure 5D), IKK (Figure 5E), p-MEKK (Figure 5F), p-JNK (Figure 5G), ASK-1 (Figure 5H), caspase-3 (Figure 5I), and NF-κB (Figure 5J) in MSShG and SSShG groups (*p* < 0.05), suggested STC-1 reduces the protein level of PKC-α (Figure 5B), p-ERK (Figure 5C), p53 (Figure 5D), IKK (Figure 5E), p-MEKK (Figure 5F), p-JNK (Figure 5G), ASK-1 (Figure 5H), caspase-3 (Figure 5I), and NF-κB (Figure 5J). All the results suggest that the variance of STC-1 can affect the phosphate modification of some key enzymes, which are closely related with the functions of these proteins. Comparatively, the changes for the ratios of non-phosphate proteins and phosphate protein were significant in JNK, ERK and MEKK but not for IKK (*p* < 0.05) (Figure 5K). STC-1 significantly affects the phosphate situation of JNK, ERK and MEKK.

## 3. Discussion

Present findings suggest that overexpression of STC-1 repairs the injury of RIRI mouse model from three main aspects: improving the anti-oxidant activity of RIRI mouse model by improving the levels of SOD and reducing MDA level; increasing the anti-inflammation activity of RIRI mouse model by reducing the concentrations of IL-6 and IFN-γ; and inhibiting the apoptosis activity of RIRI mouse model by decreasing the levels of p53 and capase-3. However, many of these functions cannot performed by STC-1 alone since it can affect the levels of many other cytokines. Thus, present work mainly focuses on the relationship between STC-1 and multiple pathways.

Much work showed that STC-1 has protective functions for normal physiological activity of cells [13] as an anti-inflammatory factor [13,15,37,38]. In macrophages, STC-1 affects macrophage mobility by inhibiting intracellular calcium signaling pathway or suppressing the generation of superoxide species [39]. Other research showed that STC-1 inhibited inflammatory signaling by affecting the levels of nuclear factor NF-κB and Jun N-terminal kinase [40]. All of these results suggest there are more other molecular mechanisms for anti-inflammatory activity of STC-1 and a number of novel mechanisms need to be explored to understand the functions of STC-1 well.

**Figure 6:**
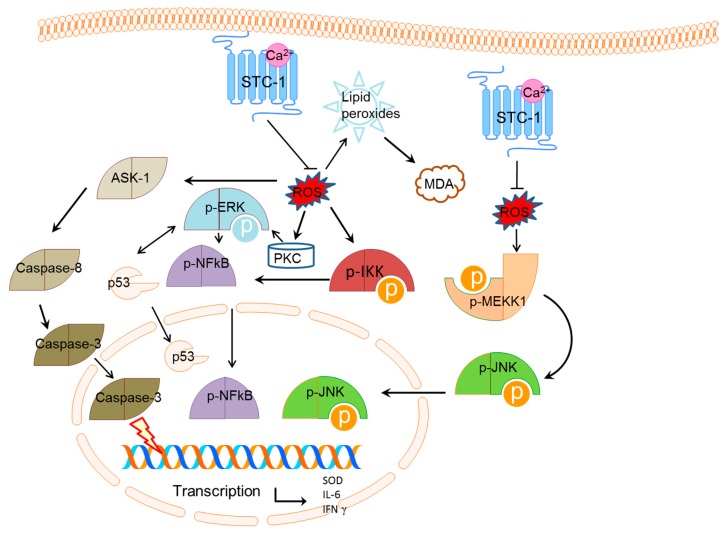
STC-1 acts a modulator for renal injury via affecting ROS-mediated multiple pathways. STC-1 is an effective ROS scavenger, which affects the levels of phosphorylated mitogen-activated protein kinase kinase kinase (p-MEKK-1), c-Jun N-terminal kinase (p-JNK), nuclear factor (NF) κB, extracellular signal-regulated kinase (p-ERK), IkB kinase (p-IKK), apoptosis signal-regulating kinase 1 (ASK-1), p53 and caspase-3. STC-1 affects ROS-mediated IKK-NF-κB, PKC-ERK-NF-κB, ASK-1-NF-κB, p53 and MEKK-JNK pathways, and improves anti-inflammation, anti-oxidant and anti-apoptosis activities by increasing level of superoxide dismutase (SOD) and reducing the level of capase-3, p53, interleukin-6 (IL-6) and IFN-γ.

Present results showed that STC-1 affected at least four ROS-mediated signaling pathways (Figure 6).

(1) Hypoxic condition inhibits ROS generation and intracellular Ca^2+^ overload [41] while overexpression of STC-1 reduces the level of ROS [17]. STC-1 inhibits the Ca^2+^ level by controlling ROS production (Figure 6). Meanwhile, the reduce of Ca^2+^ level controls ASK-1- and caspase-3-induced apoptosis [42]. Therefore, STC-1 prevents the development of kidney apoptosis by affecting ROS-mediated ASK-1 pathway [43].

(2) PKC deletion has been reported to increase the survival rate and to attenuate the kidney injury [44]. However, present results showed that the level of PKC increased when STC-1 was overexpressed, and the level of PKC increased when *STC-1* gene was blocked (Figure 4 and Figure 5). ERK-1 signaling pathway plays an important role in the balance for the cell death and proliferation. ERK-1 is necessary for repairing the damage of tubular epithelial cells and inhibiting the fibrosis caused by renal injury [45]. Here, we also found that the overexpression of STC-1 reduced the level of ERK-1 (Figure 4 and Figure 5). For NF-κB, it has been widely report to accelerate the renal injury [46,47]. Toll-like receptor 4 can mediate inflammatory responses after renal injury. AT13387 is an inhibitor of heat shock protein 90, which can repair renal injury with low toxicity. AT13387 treatment can inhibit the activity of NF-κB, which can abolish Toll-like receptor 4-mediated NF-κB activity via hyaluronan. AT13387 is a potential drug for repressing the inflammatory activity caused by NF-κB [47]. Our results indicated that the overexpression of STC-1 decreased the level of NF-κB while the deletion of STC-1 increased the level of p-NF-κB (Figure 4D and Figure 5D). The result accelerates the repair of RIRI. PKC/ERK/NF-κB pathway promotes cell apoptosis by affecting the level of p53 [48]. Thus, STC-1 prevents the development of kidney cell apoptosis by affecting ROS-mediated PKC/ERK/NF-κB pathways (Figure 6).

(3) JNK and its upstream activator p-MEKK-1, stress-activated kinase, regulate cell growth and apoptosis [49]. Previous work showed that aloeemodin-mediated photodynamic therapy induced the autophagy and apoptosis of human cells by activating ROS-JNK signaling pathway [50]. Present results showed that the increase of STC-1 reduced the level of PMEKK-1 and JNK (Figure 4, Figure 5 and Figure 6), suggesting that STC-1 affects cell apoptosis by affecting ROS-mediated MEKK-JNK pathway (Figure 6).

(4) ROS acting as the upstream molecule of NF-κB, also affects IKK-NF-κB pathway [30]. Previous work showed that IKK expression and NF-κB secretion can increase rheumatoid arthritis synovial fibroblasts apoptosis by improving the level of IL-6 and CD147 [51]. IKK/NF-κB signaling pathway plays a critical role in the tissue injury by affecting cell apoptosis [52]. Thus, IKK-NF-κB pathway is associated with the cell apoptosis and inflammation. Present results showed that the increase of STC-1 reduced the level of IKK and NF-κB (Figure 4, Figure 5 and Figure 6), suggesting that STC-1 affects cell apoptosis and inflammation by affecting ROS-mediated IKK-NF-κ pathways (Figure 6).

Moreover, our results showed that STC-1 overexpression and knockout affected the phosphate-modification ERK, IKK, JNK, and MEKK-1, which was seldom reported in the molecular mechanism for RIRI. Many data showed that NF-κB and JNK pathways are involved with the expression of IL-6 [53], while ERK and JNK pathways are involved with the expression of IFN [54]. SOD expression suppressed JNK and p38 phosphorylation, and attenuated intracellular injury [55]. These results suggest that STC-1 protects a mouse model from RIRI by affecting SOD-mediated multiple pathways and increasing anti-oxidant, anti-apoptosis and immune activity in kidney cells.

Although many molecules were investigated, present work was still limited. Many interactions among these important molecules involving in the progression of renal injury were not considered here. The molecules affecting the expression of IL-6, IFN-γ and SOD were not explored either. On the other hand, all ROS-mediated pathways are not independent and they affect each other. Finally, the detail molecular mechanism remains unknown although the modification is an important factor for the activities of these molecules. Therefore, much work needs to be done to confirm the mechanism in detail.

## 4. Materials and Methods

### 4.1. Materials

A plasmid shuttle for packaged Adeno-associated virus (AAV), pSNAV, was purchased from AGTC Gene Technology (Beijing, China). Lentiviral vector pLL3.7 was purchased from Shanghai CPG Biotech Co., Ltd. (Shanghai, China). ELISA for stanniocalcin-1 (STC-1) (Production No. SEC874Mu) ELISA Kit for mouse p53 (Product No., SEA928Mu), ELISA Kit for mouse caspase 3 (Product No., SEA626Mu), ELISA Kit for mouse interleukin-6 (Production No., SCA079Mu) and ELISA Kit for mouse IFN-γ (Production No., SCA049Mu), were purchased from Wuhan USCN Business Co., Ltd. (Wuhan, China). The following antibodies were purchased from Santa Cruz Biotech (Dallas, TX, USA): Rabbit anti-mouse STC-1 antibody (Cat. No. sc-30183, dilution 1:1000), rabbit anti-mouse NF- κB antibody (Cat. No. sc-372, dilution 1:1000), rabbit anti-mouse p53 antibody (Cat. No. sc-1311-R, dilution 1:1000), rabbit anti-mouse ASK-1 antibody (Cat. No. sc-7931, dilution 1:1000), rabbit anti-mouse caspase-3 antibody (Cat. No. sc-7148, dilution 1:1000), rabbit anti-mouse p-MEKK-1 antibody (Cat. No. sc-130202, dilution 1:1000), mouse anti-mouse p-MEKK-1 antibody (Cat. No. sc-219, dilution 1:1000), rabbit anti-mouse p-JNK antibody (Cat. No. sc-135642, dilution 1:1000), rabbit anti-mouse JNK antibody (Cat. No. sc-571, dilution 1:1000), rabbit anti-mouse p-ERK antibody (Cat. No. sc-16982-R, dilution 1:1000), rabbit anti-mouse ERK antibody (Cat. No. sc-94, dilution 1:1000), rabbit anti-mouse p-IKK antibody (Cat. No. sc-21661-R, dilution 1:1000), rabbit anti-mouse IKK antibody (Cat. No. sc-10760, dilution 1:1000) and rabbit anti-mouse PKC antibody (Cat. No. sc-10800, dilution 1:1000), rabbit anti-mouse B-Actin antibody (Cat. No. sc-130656, dilution 1:1000) and Goat anti-rabbit horseradish peroxidase-conjugated secondary antibody (IgG-HRP, Cat. No. Sc-2004, dilution 1:5000). A total of 64 male C57BL/6 mice (25 to 35 g, four weeks) were purchased from animal center of Chongqing University (Chongqing, China).

### 4.2. Establishment of Renal Ischemia-Reperfusion Model

All protocols were conducted based on the guidance for the use of laboratory animals [56] and approved by ethics review board of China Medical University. Briefly, eight male C57BL/6 mice were anesthetized using pentobarbital at 40 mg/kg, received bilateral flank incisions and exposed the renal pedicles. Non-traumatic vascular clamps (Roboz Surgical Instrument Co., Gaithersburg, MD, USA) were used to clamp each renal pedicle for 30 min. Kidney turned into dark brown, suggesting that ischemia was established successfully. After 30-min ischemia, the clamps were removed, kidney recovered as red, suggesting that blood re-perfused successfully. Eight sham animals received the similar surgical operation but no clamps for the renal pedicles. During the surgery, mice were treated with warm lamp and saline. Mice were sacrificed after 16-h RIRI surgery, blood was taken by cardiac puncturing (the samples would be used for the assay of creatinine clearance) and kidneys were obtained for the subsequent analysis.

### 4.3. Assessment of Renal Function

For urine collection at 0, 4, 8, 12 and 16 h, mice were placed in metabolic cages for 16 h before being killed in a euthanized way. Serum creatinine was measured by capillary electrophoresis (Beijing Kaiao Technology Development Co., Ltd., Beijing, China). Urine creatinine was measured by a creatinine assay kit according to manufacturer’s instructions of Sigma (Production No. MAK079, St. Louis, MO, USA).

### 4.4. Biodices Index Assay

The serum levels of IL-6, IFN-γ, p53, capase-3 and STC-1 were measured by Mouse IL-6 ELISA Kit (The Thermo Fisher Scientific, Inc., Cat. No. EM2IL6, Waltham, MA, USA), Mouse IFN-γ ELISA Kit (The Thermo Fisher Scientific, Inc., Cat. No. EM1001, Waltham, MA, USA), Mouse p53 ELISA Kit (The Thermo Fisher Scientific, Inc., Cat. No.62216, Waltham, MA, USA), Mouse caspase-3 ELISA Kit (The Thermo Fisher Scientific, Inc., Cat. No. EM1001, Waltham, MA, USA), mouse Sandwich ELISA (enzyme-linked immunosorbent assay) kit (LifeSpan Biosciences, Inc., Seattle, Cat. No. LS-F7190, WA, USA). The activity of SOD was measured as previously described [57]. SOD activity was assessed by referring the amounts of superoxide radicals produced by xanthine and xanthine oxidase. The final products were measured using steady-state fluorescence spectrofluorometer (HORIBA (China) Trading Co., Ltd., Shanghai, China) at 525 nm. The level of malondialdehyde (MDA) was measured by using a fluorometric method [57]. Briefly, 50-µL MDA buffer was added to ten-mL glass tubes with one-mL ddH_2_O. Final 29 mmol/L thiobarbituric acid was added and mixed. All samples were incubated with water at 98 °C for one h. The mixture was placed on ice for 10 min and 20-µL five M HCl was used, and agitated for ten min by adding 4-mL n-butanol. The butanol phase was separated by centrifugation at 2000 *g* for 10 min. The absorbing values were measured by using the same steady-state fluorescence spectrofluorometer.

### 4.5. Isolation of Renal Progenitor Cells from Renal Cortex of Mouse

Renal progenitor cells were isolated from the renal cortex of model and sham mice after 16-h RIRI surgery. Kidney was perfused via the aorta with the buffer (50 mM PBS and 80 U/mL heparin). Renal capsules were removed by using forceps. Isolated renal tissues were immersed in ice-cold DMEM medium. The tissues were was sliced and homogenized into 1 mm^3^ pieces, and resuspended in collagenase type IV solution (Collagenase type IV 1 mg/mL), Deoxyribonuclease 0.1 mg/mL, Bovine serum albumin (BSA) 1 mg/mL, and all regents were from Sigma Chemical Co (St. Louis, MO, USA). The mixture was cultured at 37 °C in with 100-rpm shaking for 15 min. The mixture was homogenized by pipetting ten times through a sterile transfer pipette. One-milliliter collagenase type IV solution was added and whole procedure was repeated three times. A total of 20-mL DMEM medium was added into the digestive solution. The suspension was centrifuged at 200 *g* for 5 min. The pellets were resuspended and washed by DMEM medium for three times. Renal progenitor cells were collected via density-gradient centrifugation of 45% (*v*/*v*) sterile Percoll solution. Isolated cells were washed three times with cold DMEM for next step.

### 4.6. Assay of Intracellular ROS

Intracellular ROS were measured using Dichloro-dihydro-fluorescein diacetate (DCFH-DA) assay according to previous report [58]. The isolated kidney cells were incubated with DCFH-DA (20 µM) in DMEM at 37 °C for half an hour. Subsequently, the medium was removed and the samples were washed three times by using PBS buffer. The fluorescence was measured by using a fluorescence plate reader (BMG Labtech, Ortenberg, Germany). The excitation wavelength was 485 nm and emission wavelength was 535 nm. Relative ROS generation was calculated as the percentage of fluorescence for the models over sham controls.

### 4.7. Construction of Plasmids

The recombinant adeno-associated virus pSNAV-STC-1 vectors were constructed according to a previous report [59]. Briefly, a mouse STC-1 gene was cloned by RT-PCR from mouse cDNA and inserted into pSNAV to construct pSNAV-STC-1, which encoded STC-1 under the control of the CMV promoter. The reconstructed plasmids were transferred to into NRK-52E cells using Lipofectamine 2000 (Invitrogen, Carlsbad, CA, USA). Subsequently, the confluent cells were infected by helper virus HSV1-rc/UL2 to construct rAAV2/1-STC-1 vectors. rAAV2/1-STC-1 vectors were purified by chloroform and NaCl. Chloroform (10%, *v*/*v*) was added to infected cells and incubated at 37 °C with vigorous shake until the mixture became clear, and then NaCl was added to final 1 mol/L. PEG 8000 was added to the final concentration of 8% (*w*/*v*) and centrifuged at 2000 *g* for 30 min, and the supernatant was discarded. The pellets were re-suspended in 20 mM PBS buffer (pH 8.0). An equal volume of chloroform was added and centrifuged at 5000 *g* for 30 min. The aqueous phases with the rAAV-STC-1 vectors were collected, aliquoted in high titer (10^12^ to 10^13^ particles/mL) and stored at −80 °C. NRK-52E cells were maintained in DMEM medium (Invitrogen, Carlsbad, CA, USA) with 10% FBS (Invitrogen, Carlsbad, CA, USA) and 2 mM l-glutamine. All the cells were cultured at 37 °C with 5% CO_2_.

### 4.8. Construction of STC-1 shRNA

ShRNA STC-1 was cloned into the XhoI and Hap I sites of pLL3.7 vector and packaging plasmids pRSVRev, pMDLgpRRE, and pMD.G were used for the third-generation lentiviral vector reconstruction. NRK-52E cells were cultured in flasks, trypsinized and seeded into flasks on the day before transduction. Lentiviral-shRNA was diluted to ten lentiviral particles/cell. After 48 h, the transduced cells were harvested and lentiviral-STC-1-shRNA was purified and diluted to 7.0 × 10^6^ particles/μL.

### 4.9. Groups

**Figure 7:**
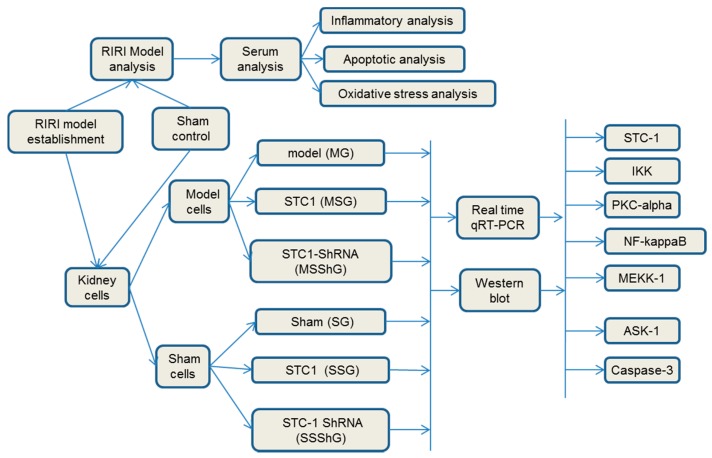
The flowchart of the study. Among 16 mice, eight mice were used to create RIRI models as a model group and another eight mice were used as a sham group. The RIRI model was evaluated by comparing the creatine clearance rate between RIRI models and sham controls. The serum levels of interleukin-6 (IL-6), interferon (IFN) γ, P53, capase-3, superoxide dismutase (SOD) and malondialdehyde (MDA) were measured in these mice. Renal progenitor cells were isolated from the kidney cortex of RIRI model and sham mice after 16-h surgery. For the kidney cells isolated from eight model mice, they were further subdivided into three groups based on different treatment options (model group (MG); STC-1 expression group (MSG), the mice were transfected with STC-1 gene to overexpress STC-1; and STC-1 shRNA group (MSShG), the mice were transfected with STC-1 shRNA to knockdown STC-1). In the same way, the kidney cells from 32 sham mice were subdivided into another three groups based on different treatment options (sham group (SG); STC-1 expression group (SSG), the mice were transfected with *STC-1* gene to overexpress STC-1; and STC-1 shRNA group (SSShG), the mice were transfected with STC-1 shRNA to knockdown STC-1). Real-time quantitative PCR and Western blot analysis analyzed the levels of Stanniocalcin-1 (STC-1), apoptosis signaling kinase (ASK-1), protein kinase C (PKC-α), phosphate mitogen-activated protein kinase kinase kinase (p-MEKK-1), MEKK-1, phosphate c-Jun N-terminal kinase (p-JNK), JNK, nuclear factor (NF) κB, phosphate extracellular signal-regulated kinase (p-ERK), ERK, phosphate IkB kinase (p-IKK), IKK, and caspase-3.

The RIRI model was evaluated by comparing the creatine clearance rate between RIRI models and sham controls. Isolated renal progenitor cells from sham and model mice were transfected with rAAV-STC-1 or 2-μL lentiviral-STC-1-shRNA particles. The mutant cells were further cultured in DMEM medium for 24 h. As Figure 7 showed, Among 16 mice, eight mice were used to create RIRI models as a model group and another eight mice were used as a sham group. Renal progenitor cells were isolated from the kidney cortex of RIRI model and sham mice after 16-h surgery. For the kidney cells isolated from eight model mice, they were further subdivided into three groups based on different treatment options (model group (MG); STC-1 expression group (MSG), the mice were transfected with *STC-1* gene to overexpress STC-1; and STC-1 shRNA group (MSShG), the mice were transfected with STC-1 shRNA to knockdown STC-1). In the same way, the kidney cells from 8 sham mice were subdivided into another three groups based on different treatment options: sham groups (sham group (SG); STC-1 expression group (SSG), the mice were transfected with *STC-1* gene to overexpress STC-1; and STC-1 shRNA group (SSShG), the mice were transfected with STC-1 shRNA to knockdown STC-1).

### 4.10. qRT-PCR (Real-Time Reverse Transcription-PCR)

Total RNA from kidney cells was isolated using TaKaRa MiniBEST Universal RNA Extraction Kit (TaKaRa Biotechnology (Dalian) Co., Ltd., Dalian, China). One-ug RNA from each sample was used for cDNA synthesis using TaKaRa RNA PCR Kit (AMV) Ver.2.1 (TaKaRa Biotechnology (Dalian) Co., Ltd., Dalian, China). QPCR was performed using the primers: STC-1, sense 5’-gaagtggttcgctgcctcaa-3’, antisense 5’-tgagtgtcaaatttagcagc-3’ (140 bp); PKC-α, sense 5’-gcccgcttcttcaagcaacc-3’, antisense 5’-ggacaagagaacgtaacgaa-3’ (160 bp); ERK, sense 5’-ctcaagatctgtgactttgg-3’, antisense 5’-ccaaatatcaatggacttgg-3’ (210 bp); p53, sense 5’-atggacgatctgttgctgcc-3’, antisense 5’-gagaagggacaaaagatgac-3’ (169 bp); NF-κB, sense 5’-taccttcaaatattagagca-3’, antisense 5’-tagttgcaaattttgacctg-3’ (140 bp); IKK, sense 5’-aacactaccagctacctgcg-3’, antisense 5’-ggctcccactggagcagtac-3’ (160 bp); MEKK-1, sense 5’-cggcagctgcgcaaagtgcg-3’, antisense 5’-tcggtaaggtgggcgccgcg-3’ (200 bp); JNK, sense 5’-agcaggaaggagcgtcccac-3’, antisense 5’-ctggagctggactgtagctg-3’ (140 bp); ASK-1, sense 5’-cgccctttgcgtccgtgggc-3’, antisense 5’-gcgctctccacgttccagaa-3’ (160 bp); caspase-3, sense 5’-gaagatgatgagacagaggc-3’, antisense 5’-tcttctgagcaaatgtctcc-3’ (140 bp); β actin: sense 5’-ttcccctccatcgtgggccg-3’, antisense 5’-gtcccagttggtaacaatgc-3’.

### 4.11. Western Blotting

All samples were suspended in RIPA buffer, homogenized by a homogenizer (Beijing Kwinbon Biotechnology CO., Ltd., Beijing, China) and centrifuged at 10,000 *g* for 30 min to remove tissue debris. The proteins in the mixture solution were separated by 12% SDS-PAGE, transferred to nitrocellulose membrane and incubated with the first antibodies for STC-1, ASK-1, PKC-α, p-ERK, ERK, p53, p-IKK, IKK, p-MEKK-1, MEKK-1, p-JNK, JNK, NF-κB, Casepase-3 and B actin. After wash with PBS containing 0.1% Tween-20, the membrane was incubated with IgG-HRP. The target proteins were viewed by chemiluminescence. The densitometric analysis was conducted by NIH ImageJ Software (National Institutes of Health, Bethesda, MA, USA).

### 4.12. Statistical Analysis

Data are expressed as means ± S.D. and are compared by one way analysis of variance (ANOVA) for different groups. Statistical significance of difference was defined by a *p*-value of less than 0.05.

## 5. Conclusions

STC-1 affected ROS-mediated ASK-1, IKK-NF-κB, PKC-ERK-NF-κB and MEKK-JNK pathways, especially through phospho-modifications of the respective proteins, resulting in the enhancement of anti-inflammation, anti-oxidant and anti-apoptosis activities, probably by affecting the levels of SOD, capase-3, p53, IL-6 and IFN-γ. The improvement of these activities may be the main molecular mechanisms for enhancing ischemic tolerance caused by RIRI, suggesting that STC-1 plays an important role in reducing RIRI by affecting ROS-mediated multiple pathways. To make sure of the conclusion, much work still needs to be done in the future.

## Abbreviations

The following abbreviations are used in this manuscript:

ASK-1	apoptosis signal-regulating kinase 1
STC-1	stanniocalcin-1
RIRI	renal ischemia-reperfusion injury
ROS	reactive oxygen species
IL-6	interleukin-6
SOD	superoxide dismutase
MDA	malondialdehyde
IFN	interferon
p-MEKK-1	phosphorylated mitogen-activated protein kinase kinase kinase
JNK	c-Jun N-terminal kinase
NF	nuclear factor
ERK	extracellular signal-regulated kinase
IKK	IkB kinase
